# Sequential action of *FRUITFULL* as a modulator of the activity of the floral regulators *SVP* and *SOC1*

**DOI:** 10.1093/jxb/ert482

**Published:** 2014-01-24

**Authors:** Vicente Balanzà, Irene Martínez-Fernández, Cristina Ferrándiz

**Affiliations:** Instituto de Biología Molecular y Celular de Plantas, Consejo Superior de Investigaciones Científicas–Universidad Politécnica de Valencia, Avenida de los Naranjos s/n, 46022 Valencia, Spain

**Keywords:** Flowering, FUL, SVP, SOC1, FLC, MADS-box factors.

## Abstract

The role in flowering time of the MADS-box transcription factor *FRUITFULL* (*FUL*) has been proposed in many works. *FUL* has been connected to several flowering pathways as a target of the photoperiod, ambient temperature, and age pathways and it is has been shown to promote flowering in a partially redundant manner with *SUPPRESSOR OF OVEREXPRESSION OF CONSTANS 1* (*SOC1*). However, the position of *FUL* in these genetic networks, as well as the functional output of FUL activity during floral transition, remains unclear. In this work, a genetic approach has been undertaken to understand better the functional hierarchies involving *FUL* and other MADS-box factors with well established roles as floral integrators such as *SOC1, SHORT VEGETATIVE PHASE* (*SVP*) or *FLOWERING LOCUS C* (*FLC*). Our results suggest a prominent role of FUL in promoting reproductive transition when photoinductive signalling is suppressed by short-day conditions or by high levels of *FLC* expression, as in non-vernalized winter ecotypes. A model is proposed where the sequential formation of FUL–SVP and FUL–SOC1 heterodimers may mediate the vegetative and meristem identity transitions, counteracting the repressive effect of FLC and SVP on flowering.

## Summary

FRUITFULL is uncovered as a modulator of the function of flowering time integrators, highlighting the importance of protein–protein complexes in the fine-tuning of the flowering time response.

## Introduction

*Arabidopsis thaliana* adult life cycle comprises three major phase transitions that are mainly characterized by the identity of the lateral structures produced by the shoot apical meristem (SAM). The vegetative phase transition marks the change from the production of juvenile leaves to the production of adult leaves. Both types of leaves form a rosette through the period of vegetative growth of the plant and, then, triggered by both environmental and endogenous cues, the SAM undergoes two subsequent phase transitions leading to reproductive development: the reproductive transition that causes bolting of the primary inflorescence and the production of cauline leaves subtending secondary inflorescences, and the meristem identity transition, after which the SAM will produce floral meristems directly (Araki, 2001; Yamaguchi *et al.*, 2009; Huijser and Schmid, 2011).

Both reproductive and meristem identity transitions, that are collectively named as floral transition, are highly controlled by developmental and environmental signals. Six promoting pathways have been proposed to regulate this process (reviewed in Fornara *et al.*, 2010; Srikanth and Schmid, 2011): the photoperiod, vernalization, ambient temperature, age, autonomous, and gibberellin pathways. The first three pathways respond to environmental signals such as daylength and seasonal or day growth temperature, while the age and autonomous patways respond to endogenous signals, and the gibberellin pathway responds to both environmental and endogenous clues. All these pathways converge at the level of a few genes, named floral transition integrators.

Within this group of floral transition integrators, several members of the MADS-box family have major roles: the expression of *SUPPRESSOR OF OVEREXPRESSION OF CONSTANS 1* (*SOC1*) is activated by the photoperiod, age and gibberellin pathways to promote floral transition (Borner *et al.*, 2000; Lee *et al.*, 2000; Samach *et al.*, 2000; Lee and Lee, 2010) which is, in part, mediated by the activation of the floral identity gene *LEAFY* (*LFY*) (Lee *et al.*, 2008; Liu *et al.*, 2008). Conversely, *FLOWERING LOCUS C* (*FLC*) and *SHORT VEGETATIVE PHASE* (*SVP*) act as floral transition repressors (Hartmann *et al.*, 2000; Michaels and Amasino, 1999; Sheldon *et al.*, 1999). High levels of *FLC* expression compete the inductive floral signals at the SAM, and thus, flowering is promoted when the vernalization and autonomous pathways repress *FLC* expression (Michaels and Amasino, 1999; Lee *et al.*, 2000; Sheldon *et al.*, 1999, 2000; Hepworth *et al.*, 2002; Michaels *et al.*, 2004; Kim *et al.*, 2009). Likewise, the expression of the flowering repressor *SVP* is controlled by the autonomous, thermosensory, and gibberellin pathways (Lee *et al.*, 2007; Li *et al.*, 2008). FLC and SVP are able to form heterodimers that directly bind to the *SOC1* promoter to down-regulate *SOC1* expression, as well as to other floral transition integrators such as *FLOWERING LOCUS T* (*FT*) (Lee *et al.*, 2007; Fujiwara *et al.*, 2008; Li *et al.*, 2008).

The MADS-box transcription factor *FRUITFULL* (*FUL*), a closely related gene to the flower meristem identity genes *APETALA1 (AP1)* and *CAULIFLOWER*, has been associated with several developmental processes. In addition to its well-known function during fruit development, *FUL* roles in floral meristem identity specification, shoot maturation, and the control of floral transition have also been described (Hempel *et al.*, 1997; Gu *et al.*, 1998; Ferrándiz *et al.*, 2000*a*, *b*; Melzer *et al.*, 2008; Shikata *et al.*, 2009; Wang *et al.*, 2009).

FUL is partially redundant with SOC1 in flowering promotion. Although the *ful* mutants are only slightly late flowering under long-day growth conditions (Ferrándiz *et al.*, 2000*a*), the double *ful soc1* mutants show a strong delay in floral transition (Melzer *et al.*, 2008). As *SOC1*, *FUL* is one of the earliest responsive genes to photoinductive signals (Hempel *et al.*, 1997; Schmid *et al.*, 2003) being a target of the FT–FD dimer (Schmid *et al.*, 2003; Teper-Bamnolker and Samach, 2005; Torti *et al.*, 2012). *FUL* also responds to signals derived from the age pathway, being one of the most responsive genes to the SQUAMOSA PROMOTER BINDING LIKE (SPL) proteins (Shikata *et al.*, 2009; Wang *et al.*, 2009; Yamaguchi *et al.*, 2009). A recent study also places *FUL* in the promotion of flowering in response to ambient temperature through the action of miR156/SPL3 and FT (Kim *et al.*, 2012).

In spite of mounting evidence linking *FUL* to the main flowering pathways, the importance of FUL in controlling these processes, as well as its position, downstream effectors, and mode of action in these pathways are still unclear. In this study, genetic analyses have been used to understand better the regulatory hierarchies involving *FUL* and other floral integrators of the MADS-box family such as *SOC1*, *SVP*, and *FLC* in the control of floral transition in Arabidopsis. Our results show that FUL is able to act both upstream and co-operatively with SOC1, forming a heterodimer and binding directly to the *LFY* promoter. In addition, it is shown that the promotive effect of FUL on floral transition depends of the presence of a functional allele of *SVP* and that FUL is able to counteract the repressive effect of FLC on flowering both affecting *FLC* expression levels and probably competing with FLC for common targets. Taking all these data together, a dynamic model is proposed for the role of FUL during floral transition, where the progressive formation of different heterodimers of FUL and other MADS transcription factors may act as a molecular switch between the vegetative and reproductive states.

## Materials and methods

### Plant material and growth conditions

*Arabidopsis thaliana* plants were grown in cabinets at 21 °C under LD (16h light) or SD (8h light) conditions, illuminated by cool-white fluorescent lamps (150 µE m^–2^ s^–1^), in a 1:1:1 by vol. mixture of sphagnum:perlite:vermiculite. To promote germination, seeds were stratified on soil at 4 °C for 3 d in the dark. The *Arabidopsis* plants used in this work were in the Col-0 background, except *ful-1* and 35S::SOC1, that were in L*er*. Mutant alleles and transgenic lines have been previously described: *soc1-2* (Lee *et al.*, 2000), *ful-1* (Gu *et al.*, 1998), *ful-2* (Ferrándiz *et al.*, 2000*a*), *svp-32* (Lee *et al.*, 2007), FRI FLC (Lee and Amasino, 1995), 35S::SOC1, (Lee *et al.*, 2000), 35S::FUL (Ferrándiz *et al.*, 2000*b*), 35S::SVP (Masiero *et al.*, 2004), 35S::FLC (Michaels and Amasino, 1999), LFY:GUS (Blázquez *et al.*, 1997) and FLC:GUS (Sheldon *et al.*, 2002).

35S::FUL::GFP was generated by cloning the FUL CDS into the pEarley103 vector (Earley *et al.*, 2006). *Agrobacterium* strain C58 pM090 was used to transform *Arabidopsis* using the floral dip protocol (Clough and Bent, 1998), and transgenic lines carrying a single transgene insertion and with similar phenotypes to the reference 35S::FUL line were selected.

### Flowering time measurements

Flowering time was scored as number of leaves at bolting. The number of rosette and cauline leaves was counted when the bolting shoot had produced the first open flower. At least 15 genetically identical plants were used to score flowering time of each genotype. The Student’s *t*-test was used to test the significance of flowering time differences.

### Chromatin immunoprecipitation (ChIP)

35S::FUL and 35S::FUL::GFP seeds were grown for 15 d in soil and inflorescences were collected for analysis. The ChIP experiments were performed as previously described by Sorefan *et al.* (2009) with minor modifications using an anti-GFP antibody (Abcam, Ab290). Q-PCR was performed using the SYBR®Green PCR Master Mix (Applied Biosystems) in a ABIPRISM 7700 sequence detection system (Applied Biosystems). The values correspond to the ratios between the pull-down DNA with the GFP antibody from 35S::FUL and 35S::FUL:GFP lines and between a 10% fraction of the input genomic DNA from both samples, all of them initially normalized by ACT7 or UBQ10 genomic region. The primers used for this study are described in Supplementary Table S1 at *JXB* online.

### Quantitative RT-PCR (qRT-PCR)

Total RNA was extracted from whole plants with the RNeasy Plant Mini kit (Qiagen). 2 µg of total RNA were used for cDNA synthesis performed with the First-Strand cDNA Synthesis kit (Invitrogen) and the qPCR master mix was prepared using the iQTM SYBR Green Supermix (Bio-Rad). Results were normalized to the expression of the *TIP41-like* reference gene. The PCR reactions were run and analysed using the ABI PRISM 7700 Sequence detection system (Applied Biosystems). Three technical and two biological replicates were performed for each sample. See Supplementary Table S1 at *JXB* online for the primer sequences.

### β-Glucuronidase (GUS) staining and activity measurements

For GUS histochemical detection, samples were treated for 15min in 90% ice-cold acetone and then washed for 5min with washing buffer (25mM sodium phosphate, 5mM ferrocyanide, 5mM ferricyanide, and 1% Triton X-100) and incubated from 4–16h at 37 °C with staining buffer (washing buffer+1mM X-Gluc). Following staining, plant material was fixed, cleared in chloral hydrate, and mounted to be viewed under bright-field microscopy.

For quantitative measurements, the protocol described in Blazquez *et al.* (1997) was followed. Briefly, apices were incubated at 37 °C for 16h in 1mM MUG assay solution (1mM 4-methyl umbelliferyl glucuronide, 50mM sodium phosphate buffer pH 7, 10mM EDTA, 0.1% SDS, 0.1% Triton X-100), in individual wells of a microtitre plate. After the reaction had been stopped by the addition of 0.3M Na_2_CO_3_, fluorescence at 430nm was measured on a luminescence spectrophotometer equipped with an ELISA plate reader (Perkin Elmer, model LS50B).

### Bimolecular Fluorescence Complementation (BiFC)

Open reading frames of full-length *FUL*, *SOC1*, and *SVP* CDS were cloned into vectors pYFPN43 and pYFPC43 (http://www.ibmcp.upv.es/FerrandoLabVectors.php), and BiFC was performed as previously described by Belda-Palazon *et al.* (2012).

### Confocal microscopy

Confocal microscopy was performed using a Leica TCS SL (Leica Microsystems GmbH, Heidelberg, Germany) equipped with an Argon krypton laser (Leica).

### Accession numbers

Sequence data from this article can be found in the *Arabidopsis* Genome Initiative or GenBank/EMBL databases under the following accession numbers: FUL (AT5G60910), SOC1 (AT2G45660), SVP (AT2G22540), FLC (AT5G10140), FRI (AT4G00650), LFY (AT5G61850), UBQ10 (AT4G05320), act7 (AT5G09810), and tip41-like (AT4G34270).

## Results

### Genetic interactions of *FUL* and *SOC1*

The timing of both reproductive and meristem phase transitions were compared by the quantification of rosette and cauline leaves of wild-type, *ful*, and 35S::FUL plants. As previously reported, it was observed that the loss of *FUL* function caused a small delay in flowering time both in long-day (LD) and short-day (SD) conditions, while the over-expression of *FUL* caused a strong early flowering phenotype (Table 1) (Ferrándiz *et al.*, 2000*a*; Melzer *et al.*, 2008). The late flowering phenotype of *ful* mutants mainly affected the onset of the meristem identity transition, since the number of rosette leaves did not significantly differ from the wild type, while the number of cauline leaves was increased in both LD and SD conditions (Table 1). In addition, when grown in SD, the axillary meristems of cauline leaves of single *ful-2* mutants formed aerial rosettes (see Supplementary Fig. S1 at *JXB* online), and flowers were subtended by bracts (see Supplementary Fig. S1 at *JXB* online).

**Table 1.** Genetic interaction of FUL and SOC1: effect on flowering

	Long day		Short day	
	*Rosette leaves*	Cauline leaves	*Rosette leaves*	Cauline leaves
Columbia-0	10.2±1.0	3.2±0.4	55.1±3.4	9.3±0.7
*ful-2*	10.7±0.8	4.4±0.5^a^	59.9±3.8^a^	23.7±3.2^a^
*soc1-2*	19.3±0.9^a^	4.2±0.5^a^	75.0±4.2^a^	15.2±0.5^a^
*ful-2 soc1-2*	24.5±0.8^a,b,c^	9.7±1.9^a.b,c^	75.1±3.5^a,b,^	28.1±1.7^a,b,c^
35S::FUL	3.5±0.5^a^	1.7±0.7^a^	10.6±0.9^a^	3.6±0.7^a^
35S::FUL *soc1-2*	9.0±1.1^d^	2.2±0.7^d^	44.6±12.8^d^	7.2±4.5^d^
Landsberg *er*	7.3±0.5	1.8±0.4	nd	nd
*ful-1*	8.4±0.5^e^	2.5±0.5^e^	nd	nd
35S::SOC1	4.0±0.0^e^	0.4±0.5^e^	nd	nd
35S::SOC1 *ful-1*	4.0±0.0^f^	0.7±0.5^f,g^	nd	nd
35S::FUL 35S::SOC1	2.0±0.0^g^	0.2±0.4^g^	nd	nd

Flowering time is expressed as the mean of rosette and cauline leaves produced in long- and short-day conditions. Errors are represented as the standard deviation. Superscript letters indicate a signiﬁcant difference (*P* <0.05) from (a) Col, (b) *ful-2*, (c) *soc1-2*, (d) 35S::FUL, (e) L*er*, (f) *ful-1*, and (g) 35S::SOC1 controls, respectively, according to Student’s *t*-test; nd=not determined.

It has been described that *FUL* and *SOC1* have similar roles and probably promote flowering redundantly (Melzer *et al.*, 2008). However, it is still unclear how precisely these two factors interact genetically and how each of them contributes to the reproductive or the meristem identity transitions. To understand better the genetic relationship of *FUL* and *SOC1*, the effect on flowering time of different combinations of *FUL* and *SOC1* loss- and gain-of-function alleles was compared.

In LD conditions, the *ful-2 soc1-2* double mutant showed a synergistic late-flowering phenotype, in agreement with previously reported data (Melzer *et al.*, 2008), producing more rosette leaves than the *soc1-2* single mutant and more cauline leaves than both *ful-2* and *soc1-2* single mutants (Table 1). Additional phenotypes were observed such as the production of small leaves subtending flowers, the development of aerial rosettes at the cauline leaf axils, and frequent SAM reversion (see Supplementary Fig. S1B at *JXB* online), similar to what was observed in *ful-2* single mutants grown in SD and in other studies (Torti *et al.*, 2012).

The *soc1-2* mutant grown in SD showed a dramatic increase in rosette leaf number, and also a delay in meristem identity transition, although not as important as the delay produced by *ful-2* (Table 1). The *ful-2 soc1-2* double mutants grown in SD produced a similar number of rosette leaves than the *soc1-2* mutant, indicating that, in the absence of photoperiodic stimulus, the promoting role of *FUL* on the reproductive transition could depend on the presence of SOC1. On the other hand, the number of cauline leaves produced by *ful-2 soc1-2* was only moderately higher than in *ful-2* single mutants, suggesting that FUL would have a predominant effect in the control of meristem identity transition (Table 1).

**Fig. 1.**
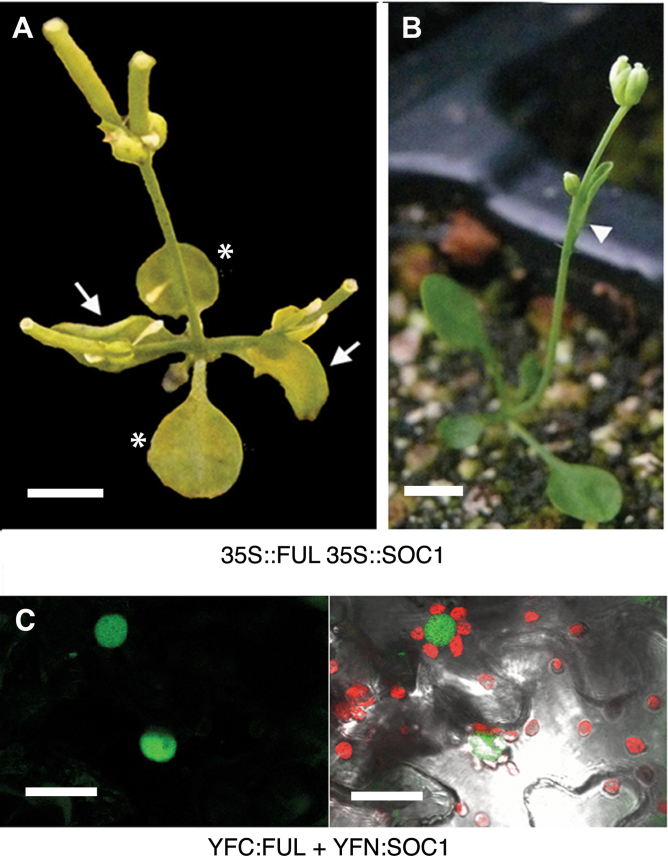
Interaction of FUL with SOC1. (A, B) Phenotypes of 35S::FUL 35S::SOC1 double over-expression lines. Only two rosette leaves are produced (arrows in A) and occasionally one cauline leaf (arrowhead in B). All axillary meristems are determinate, directly producing flowers. Asterisks mark the cotyledons in (A). (C) Bimolecular Fluorescence Complementation in tobacco epidermal leaf cells between transiently expressed FUL and SOC1 fusions to the C- and N-terminal fragments of YFP, respectively. The left panel shows reconstituted YFP fluorescence (green) and the right panel is an overlay with a bright field image of the same sector where chlorophyll is shown in red. Negative controls for BiFC experiments are shown in Supplementary Fig. S3 at *JXB* online. Scale bars: 500mm (A, B), 40 µm (C).

35S::FUL *soc1-2* plants flowered earlier than the wild type, but significantly later than 35S::FUL lines (Table 1) supporting the idea that the flowering-promoting role of FUL was partially dependent on the presence of an active allele of *SOC1*. In contrast, 35S::SOC1 *ful-1* plants were identical to 35S::SOC1 plants in rosette leaf number, while the absence of *FUL* only slightly increased the number of cauline leaves produced in the 35S::SOC1 background (Table 1). Finally, lines that over-expressed both genes simultaneously flowered extremely early, producing only two rosette leaves before the SAM directly differentiated into one or two flowers, although occasionally one cauline leaf with an axillary flower was formed (Table 1;Fig. 1A, B). Moreover, the axillary meristems from rosette leaves were also converted into flowers (Fig. 1A). This strong synergistic effect, together with the partial dependence of FUL on the presence of SOC1 to promote flowering, was compatible with FUL acting in part as an upstream regulator of *SOC1*, together with a subsequent co-operative action of both proteins in the regulation of putative common targets, although it did not exclude other possible scenarios.

### *SOC1* and *LFY* are FUL direct targets

It has been described that FUL and SOC1 are able to interact in yeast two-hybrid experiments as homo- and heterodimers (de Folter *et al.*, 2005; Immink *et al.*, 2012). To confirm this interaction *in planta*, a Bimolecular Fluorescence Complementation (BiFC) experiment was performed through transient expression on *Nicotiana benthamiana* leaves, observing FUL-SOC1 dimerization in the nuclei of the cells (Fig. 1C).

**Fig. 2.**
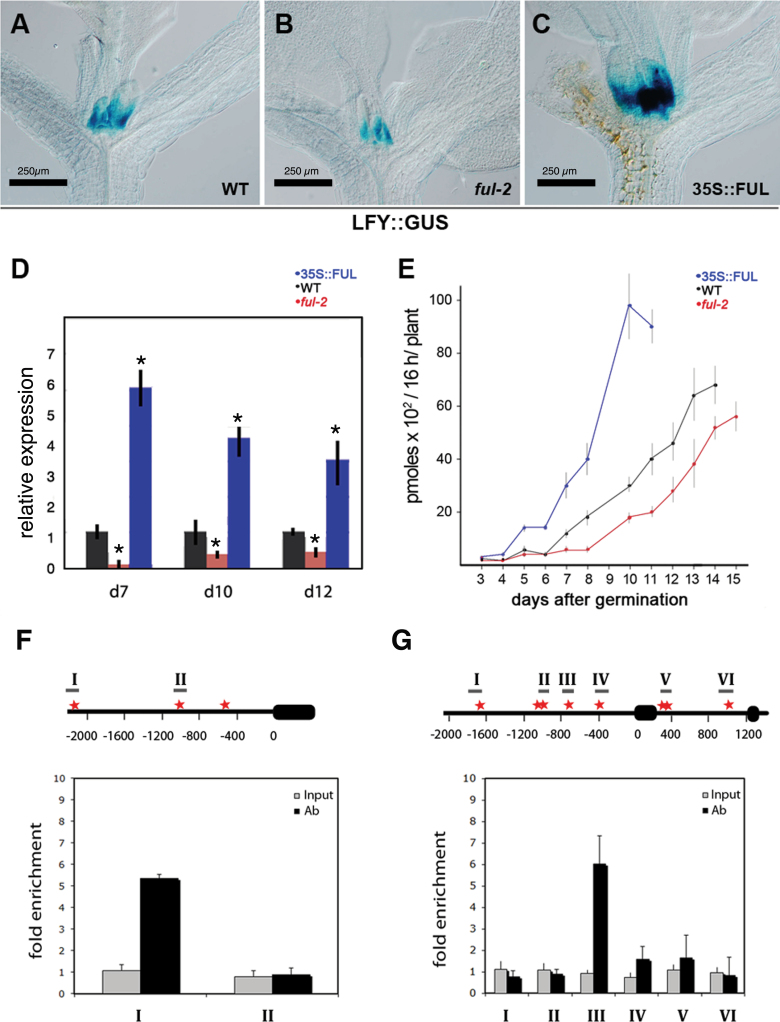
FUL regulates key genes in the floral transition process binding directly to SOC1 and LFY promoters. (A–C) Histochemical detection of LFY::GUS activity in the apices of 6-d-old wild type (A), *ful-2* (B) or 35S::FUL (C) plants. Scale bars, 250 µm. (D) Relative expression of LFY analysed by qRT-PCR in WT, *ful-2*, and 35S::FUL plants at 7, 10, and 12 d after germination. The error bars depict the s.e. based on two biological replicates. Asterisks (*) indicate a signiﬁcant difference (*P* <0.05) from the WT control according to Student’s *t*-test. (E) Quantification of LFY:GUS activity in WT, ful-2, and 35S::FUL backgrounds. Plants were grown on plates under long days (LD). At each time point, GUS activity was measured in at least 12 individual apices, and the means ±s.e are given. (F) (Top) Schematic diagram of the *LFY* upstream promoter region. First exon is represented by a black box, while the upstream genomic region is represented by a black line. The red stars indicate the sites containing either single mismatch or perfect match with the consensus binding sequence (CArG box) of MADS-domain proteins. Amplicons spanning these sites used in the ChIP analyses are represented by grey lines and marked by roman numbers. (Bottom) ChIP enrichment tests showing the binding of FUL-GFP to the LFY-I region. Bars represent the ratio of amplified DNA (35S::FUL:GFP/35S::FUL) in the starting genomic DNA (input) or in the immunoprecipitated DNA with the GFP antibody (Ab). (G) (Top) Schematic diagram of the *SOC1* genomic region, including upstream promoter, exons 1 and 2 and the first intron. Exons are represented by black boxes, upstream genomic region and intron by a black line. The red stars mark CArG boxes. Amplicons spanning these sites used in the ChIP analyses are represented by grey lines and marked by roman numbers. (Bottom) ChIP enrichment tests showing the binding of FUL-GFP to the SOC1-III region. Bars represent the ratio of amplified DNA (35S::FUL:GFP/35S::FUL) in the starting genomic DNA (input) or in the immunoprecipitated DNA with the GFP antibody (Ab).

The floral identity gene *LFY* has been identified as a bona fide SOC1 direct target (Lee *et al.*, 2008). In addition, *FUL* has been also suggested to up-regulate *LFY* (Ferrándiz *et al.*, 2000*a*). To confirm this suggestion, the expression of a LFY::GUS reporter line was analysed in the *ful-2* and 35S::FUL backgrounds, and it was observed that the level of *LFY* expression was dependent on *FUL*, being lower in the *ful-2* mutant and higher in the 35S::FUL line than in WT plants (Fig. 2A–C). These relative levels of expression were also confirmed by quantitative RT-PCR of *LFY* expression in apices at 7, 10, and 12 d after germination (Fig. 2D). In addition, GUS activity was also quantitatively determined in individual dissected apices, using the substrate 4-methyl umbelliferyl glucuronide (MUG), which is converted by GUS into the fluorescent product 4-MU. A time-course per-apex quantification was performed on the three genetic backgrounds, observing that LFY::GUS activity was consistently higher in 35S::FUL plants and lower in *ful-2* plants than in the WT (Fig 2E). Chromatin immunoprecipitations (ChIP) experiments using a 35S::FUL::GFP line (see Supplementary Fig. S2 at *JXB* online) revealed that FUL was able to bind a region 2.2kb upstream to the ATG codon of the *LFY* gene (Fig. 2F), overlapping with a previously identified region also bound by SOC1 (Lee *et al.*, 2008).

Moreover, FUL–GFP was also found to bind the *SOC1* promoter, around 800bp upstream of the ATG codon (Fig. 2G). Again, this region bound by FUL overlaps with a region bound by SOC1 itself, which confirms *in planta* the Y1H experiment reported previously, which shows a FUL–SOC1 heterodimer binding to this fragment of the SOC1 promoter (Immink *et al.*, 2012). Taken together, these results strongly support the hypothesis of SOC1 and FUL binding as heterodimers to the promoters of their target genes and could explain the genetic interactions observed.

### Genetic interactions of *FUL* and *SVP*

SVP has been shown to repress *SOC1* directly, in part by binding to the *SOC1* promoter as a heterodimer with FLC, a potent repressor of flowering involved in the vernalization and autonomous pathways (Michaels and Amasino, 1999; Sheldon *et al.*, 2002; Helliwell *et al.*, 2006). Our results indicated that FUL could also act as an upstream regulator of *SOC1*, binding directly the *SOC1* promoter. To explore whether FUL could interact with SVP to regulate *SOC1*, the effect on flowering time of different combinations of *FUL* and *SVP* loss- and gain-of-function alleles was characterized.

The *svp-32* mutant showed a clear early-flowering phenotype both in LD and SD conditions, reducing the number of rosette leaves produced when compared with the WT control, as previously described by Lee *et al.* (2007) (Table 2). *ful-2 svp-32* flowered with a similar number of leaves as the *svp-32* single mutant (Table 2) (Torti *et al.*, 2012), suggesting that SVP represses additional targets that can promote flowering in the absence of *FUL*, as has already been proposed by Torti *et al.* (2012). If this was true, we could expect plants over-expressing *FUL* in a *svp* background to flower earlier or at least like 35S::FUL plants. However, 35S::FUL *svp-32* plants also flowered similarly to *svp-32*, both in LD and SD, (Table 2) suggesting an alternative scenario where *FUL* over-expression was not able to promote flower transition in the absence of an active SVP protein. Thus, the epistatic effect of *svp* mutation on both *FUL* loss- or gain-of-function may suggest that FUL required SVP to regulate its targets, and this could be mediated by the physical interaction of both factors.

**Table 2.** Genetic interaction of FUL and SVP: effect on flowering

	Long day		Short day	
	*Rosette leaves*	Cauline leaves	*Rosette leaves*	Cauline leaves
Columbia-0	12.4±1.7	2.5±0.4	64.4±6.0	8.6±0.8
*ful-2*	12.9±0.9	3.8±0.6^a^	70.2±7.0^a^	20.8±3.8^a^
*svp-32*	5.6±0.5^a^	2.8±0.4	16.4±2.1	4.6±1.0
*ful-2 svp-32*	5.3±0.5^b^	3.3±0.5	16.1±2.5	7.1±1.6
35S::FUL	4.0±0.0^a^	1.4±0.5^a^	8.3±1.8^a^	3.5±0.8^a^
35S::FUL *svp-32*	5.8±0.4	2.5±0.5	14.9±2.1^c,d^	3.4±1.2^c^
35S::SVP	27.5±1.7^a^	7.3±1.0^a^	nd	nd
35S::FUL 35S::SVP	5.8±1.2^e^	2.7±0.8^d,e^	nd	nd

Flowering time is expressed as the mean of rosette and cauline leaves produced in long- and short-day conditions. Errors are represented as the standard deviation. Superscript letters indicate a signiﬁcant difference (*P* <0.05) from (a) Col, (b) *ful-2*, (c) *svp-32*, (d) 35S::FUL, and (e) 35S::SVP controls, respectively, according to Student’s *t*-test; nd=not determined.

**Fig. 3.**
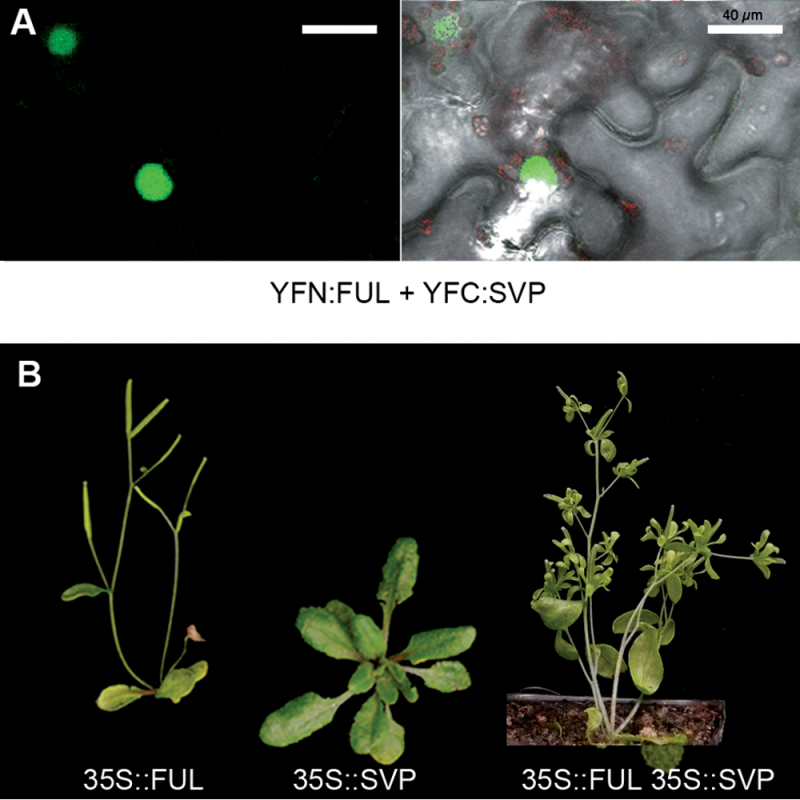
Interaction of FUL with SVP. (A) BiFC experiments in tobacco leaf cells between transiently expressed FUL and SOC1 fusions to the C- and N-terminal fragments of YFP, respectively. The left panel shows YFP reconstituted fluorescence (green) and the right panel is an overlay with a bright field image of the same sector where chlorophyll is shown in red. Negative controls for BiFC experiments are shown in Supplementary Fig. S3 at *JXB* online. Scale bars: 40 µm. (B) Phenotypes of the 35S::FUL, 35S::SVP, and 35S::FUL 35S::SVP double over-expression lines. *FUL* over-expression reverts the late flowering phenotype of 35S::SVP, although inflorescence development is partially restored respect to the 35S::FUL plants.

Interaction of FUL and SVP proteins has already been reported in yeast-two-hybrid experiments (de Folter *et al.*, 2005; Immink *et al.*, 2012). To test if this heterodimer also occurred *in planta*, a BiFC experiment was performed that confirmed such interaction (Fig 3A). If FUL required interaction with SVP to promote floral transition, it could be expected that simultaneous over-expression of FUL and SVP would result in early flowering, overcoming the late-flowering phenotype caused by SVP over-expression. A 35S::SVP 35S::FUL line was then generated and flowering time quantified in this double transgenic line. As described above, 35S::FUL flowered early, while 35S::SVP flowered very late, as expected for a potent repressor of flowering transition (Table 2; Fig. 3B). The line harbouring both the 35S::FUL and the 35S::SVP transgenes flowered early, similarly to 35S::FUL or 35S::FUL *svp* plants (Fig. 3B; Table 2). This phenotype indicated that SVP was not able to repress floral transition when both high levels of SVP and FUL were present, suggesting that the FUL–SVP dimer could suppress the repressor effect of SVP on flowering or even act as a flowering promoting factor.

### Genetic interactions of *FUL* and *FLC*

Because the repressor effect of SVP in flowering transition is partially mediated by the formation of a heterodimer with FLC (Lee *et al.*, 2007; Fujiwara *et al.*, 2008; Li *et al.*, 2008), the genetic relationship of *FUL* and FLC was studied.

**Fig. 4.**
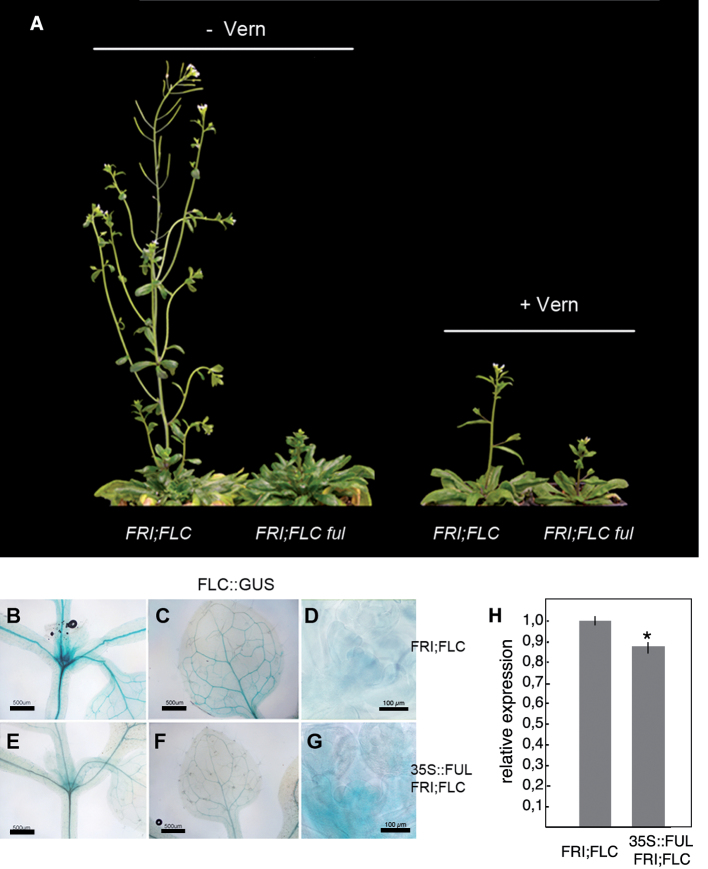
*FUL* over-expression suppresses the effects of high levels of *FLC*. (A) Vernalization response of *FRI;FLC* and FRI;FLC *ful-2* in LD. The *ful-2* mutation greatly enhances the late flowering phenotype of FRI;FLC unvernalized plants (left), while a vernalization treatment causes both genotypes to flower similarly earlier (right). (B–G) Histochemical detection of FLC::GUS activity in *FRI;FLC* (B–D) and *FRI;FLC* 35S::FUL (E–G) plants. Apices of 10-d-old plants are compared in (B) and (E), the first rosette leaf in (C) and (F), and inflorescence apices of plants at bolting in (D) and (G). All plants were heterozygous for the FLC::GUS reporter and for the wild-type dominant alleles of *FRI* or *FLC*. 35S::FUL in (E–G) was also heterozygous. Scale bars: 500 µm (B, C, E, F) or 100 µm (D, G). (H) Relative expression of *FLC* analysed by qRT-PCR in *FRI;FLC* and *FRI;FLC* 35S::FUL plants 10 d after germination. The error bars depict the s.e. based on two biological replicates. An asterisk (*) indicates a signiﬁcant difference (*P* <0.05) from the WT control according to Student’s *t*-test.

Much of the natural variation in flowering time in *Arabidopsis* depends on the allelic variation of *FLC* and its positive regulator *FRI* (Amasino, 2010). Late-flowering accessions usually bear functional alleles of both *FLC* and *FRI*, while most rapid-cycling accessions typically possess loss-of-function alleles of either gene. *ful-2* mutants are in the Col-0 genetic background, which has a *fri*;*FLC* genotype and, therefore, an early-flowering habit (Sheldon *et al.*, 1999; Johanson *et al.*, 2000; Michaels, 2009). To study the effect of *ful* mutations in the presence of *FLC*, the *ful-2* allele was introduced in a *FRI;FLC* genetic background derived from the introgression of a *FRI* functional allele into Col-0 (Lee and Amasino, 1995). *FRI;FLC* plants flower very late in all growing conditions, and are strongly responsive to vernalization treatment to induce flowering (Lee and Amasino, 1995). In LD conditions and without vernalization, the *ful-2* mutation greatly enhanced the late-flowering phenotype of *FRI;FLC* plants, as *FRI;FLC ful-2* produced many more rosette and cauline leaves than *FRI;FLC* plants (Table 3; Fig. 4A). Vernalization of both *FRI;FLC* and *FRI;FLC ful-2* significantly accelerated the reproductive transition, and both lines flowered with a similar number of rosette leaves although *FRI;FLC ful-2* still produced more cauline leaves (Table 3; Fig. 4A). Thus, vernalization significantly suppressed the effect of *ful-2* on the floral transition of *FRI;FLC* plants, suggesting that, in the presence of high levels of *FLC* (such as in non-vernalized *FRI;FLC* plants), *FUL* was required to promote flowering and that this promotion could either be mediated by negative regulation of *FLC* or by counteracting the repressor effect of *FLC* on flowering.

**Table 3.** Effect of vernalization in flowering time of *ful* mutants

	Long day			
	–Vernalization		+Vernalization	
	*Rosette leaves*	Cauline leaves	*Rosette leaves*	Cauline leaves
FRI FLC	57.6±8.0	9.5±2.2	24.4±2.1	5.9±1.0
FRI FLC *ful-2*	73.9±6.2**	19.8±0.9**	23.2±2.9	8.6±0.8

Flowering time is expressed as the mean of rosette and cauline leaves produced in long-day conditions. Errors are represented as the standard deviation. Asterisks (*) indicate a signiﬁcant difference (*P* <0.05) from the FRI FLC control according to Student’s *t*-test.

Flowering time was also analysed in plants resulting from crossing 35S::FUL to *FRI;FLC* and to 35S::FLC lines, thus generating F_1_ plants heterozygous for the *FRI* allele and hemizygous for the 35S::FUL transgene or hemizygous for both the 35S::FLC and the 35S::FUL transgenes. The results were compared with the flowering time of the corresponding F_1_s from crosses between *FRI;FLC* or 35S::FLC to the Col-0 wild type. Constitutive expression of *FUL* caused early flowering in *FRI;FLC* plants and was also able to promote flowering in the 35S::FLC background, although to a lesser extent than when *FLC* expression was controlled by its own regulatory sequences (Table 4). The activity of a FLC::GUS reporter in rosettes of 35S::FUL *FRI;FLC* plants was checked and it was found to be lower than in a *FRI;FLC* background (Fig. 4B, C, E, F). Quantitative RT-PCR showed that this reduction was modest, but significant (Fig. 4H), supporting that FUL could, at least partially, repress *FLC* expression. Moreover, while *FRI;FLC* plants only flowered when *FLC* levels were almost undetectable in the inflorescence, the 35S::FUL *FRI;FLC* plants flowered when *FLC* was still detected, indicating that FUL could also overcome the FLC repressive effect on flowering (Fig. 4D, G). Taking all these data together, it appeared that FUL was both repressing *FLC* expression and counteracting the negative effect of *FLC* on flowering, since plants were able to flower even in the presence of significant levels of FLC.

**Table 4.** Genetic interaction of FUL and FLC: effect on flowering

	Long day	
	*Rosette leaves*	Cauline leaves
**FRI/+**	56.5±1.7	12.0±1.4
**35S::FUL/+**	7.0±2.3	2.2±0.4
**35S::FLC/+**	>80	nd
**35S::FUL/+ FRI/+**	9.7±1.1^a,b^	2.3±0.8^a^
**35S::FUL/+ 35S::FLC/+**	34.3±7.7^b,c^	13.8±1.9^b^

Flowering time is expressed as the mean of rosette and cauline leaves produced in long-day conditions. Errors are represented as the standard deviation. Superscript letters indicate a signiﬁcant difference (*P* <0.05) from (a) FRI/+, (b) 35S::FUL/+, and (c) 35S::FLC/+ controls, respectively, according to Student’s *t*-test; nd=not determined.

## Discussion

**Fig. 5.**
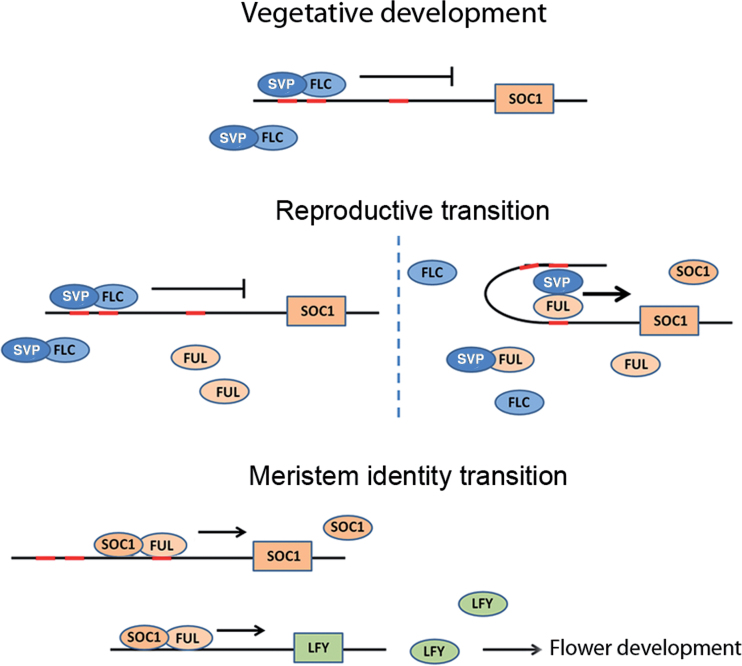
A proposed mechanistic model for the role of FUL during floral transition through interaction with SVP and SOC1 factors. During vegetative growth FLC and SVP repress the expression of *SOC1* and other flowering promoting factors. Upon FUL accumulation, probably mediated by the age SPL-dependent pathway, FUL–SVP dimerization occurs. The FUL–SVP dimer could compete with the FLC–SVP dimer for binding sites in the *SOC1* promoter and/or directly interfering with the FLC–SVP dimer formation. Lower repressive activity of the FLC-SVP dimer on *SOC1* or even direct activation of *SOC1* by FUL-SVP would lead to SOC1 accumulation, the dimerization of FUL-SOC1 and the activation of both *SOC1* and *LFY* promoters, thus triggering flower initiation.

The results presented in this study show that *FUL* participates in both reproductive and meristem identity transitions modulating the activity of MADS-box factors with major regulatory roles in these phase changes. The role of FUL in promoting meristem identity transition is co-operative and partly dependent on SOC1, while the role of FUL in reproductive transition may be mediated both by interfering with the FLC–SVP dimer and/or changing the activity of SVP from a repressor to an activator of flowering. Taking together our genetic analyses and the results from BiFC dimerization experiments, it is proposed that these regulatory interactions are probably mediated by the sequential participation of FUL in heterodimers with SVP and SOC1 (Fig. 5).

### *FUL* promotes flower initiation together with *SOC1*

Previous studies indicate that *FUL* and *SOC1* are able to act redundantly to promote floral transition. *FUL* and *SOC1* share common upstream regulators, as they are both activated by the FT–FD complex and repressed by SVP (Lee *et al.*, 2007; Li *et al.*, 2008; Torti and Fornara, 2012). However, they also respond differently to other flowering pathways, *FUL* being more responsive to the age pathway and *SOC1* to the gibberellin pathway (Wang *et al.*, 2009; Yamaguchi *et al.*, 2009; Porri *et al.*, 2012). Moreover, recent work has also shown how *SOC1* and *FUL* respond differently to the signals from the photoperiodic pathway, where the maintenance of *SOC1* expression in the SAM depends more strongly on a continuous photoperiodic stimulus than that of *FUL* (Torti *et al.*, 2012). These differences in regulation could partly explain the phenotypic effects that were observed in *ful* and *soc1* mutants. When grown in SD, *ful* mutants show little effect in reproductive transition, while strongly delaying flower production, indicating that when other photoperiod-responsive genes like *SOC1* are down-regulated, *FUL* plays an important role in promoting floral meristem initiation. Moreover, the presence of binding sites for FUL in the *SOC1* promoter, the similar timing of reproductive transition in *soc1* and *ful soc1* mutants grown in SD, and the significant suppression of the early-flowering phenotype of 35S::FUL lines in the *soc1* background, probably places *FUL* upstream of *SOC1*, suggesting that, in the absence of a photoperiodic stimulus, FUL could directly mediate the activation of *SOC1*. Moreover, previous reports on SOC1 binding to its own promoter (Immink *et al.*, 2012) and our experiments showing binding of FUL to the same region of the *SOC1* promoter also suggest that, once both factors are present, they could act in a positive feedback loop to maintain high levels of *SOC1* expression. This positive feedback loop could also explain why a *ful* mutant grown in SD, where *SOC1* expression is down-regulated, shows meristem reversion and bracts subtending flowers. On the other hand, no binding sites for SOC1 on the *FUL* promoter have been identified in a recent ChIP-seq experiment (Tao *et al.*, 2012), and loss of *FUL* function does not modify the 35S::SOC1 early flowering phenotype, suggesting that FUL is not a target of SOC1 regulation and, therefore, of this feedback loop.

Our results also show that FUL and SOC1 appear to act co-operatively in promoting a sharp meristem identity transition through the activation of *LFY*. A similar model has been proposed for the interaction of SOC1 and AGL24, another MADS factor with a flowering promoting role (Michaels *et al.*, 2003). SOC1 has been described as a cytoplasmic protein able to dimerize with AGL24, and to translocate to the nucleus to up-regulate *LFY* expression (Lee *et al.*, 2008; Li *et al.*, 2008). A similar mechanism appears to be working for FUL and SOC1, as it has been observed that FUL and SOC1 are able to dimerize in the nucleus, and that both SOC1 and FUL bind to the same region of the *LFY* promoter. Thus SOC1, AGL24 and FUL could be forming redundant dimers or a higher order molecular complex to ensure the initiation of floral meristems through *LFY* activation.

### SVP behaviour as a repressor of flowering is probably suppressed by its interaction with FUL

Because *svp* mutations largely suppress the late-flowering phenotype of *soc1* and *ful* mutants, it has been proposed that SVP represses additional flowering-promoting factors that would act in parallel to *FUL* and *SOC1* and, therefore, even in the absence of FUL and SOC1 functions, the derepression of these factors would still cause early flowering (Torti *et al.*, 2012). Our results, showing that *FUL* over-expression suppresses the strong late-flowering phenotype of *SVP* over-expression and that SVP and FUL are able to dimerize, may suggest a different interpretation. A possibility would be that *FUL* over-expression could overcome the down-regulation of these additional flowering-promoting factors repressed by SVP. However, this is in contradiction to our data showing that *soc1* mutations only partially suppress 35S::FUL early-flowering phenotypes and by the phenotype of 35S::SVP 35S::SOC1 plants, which flower earlier than 35S::SVP plants but later than 35S::SVP 35S::FUL plants (Li *et al.*, 2008). We can then speculate about the role of the SVP–FUL putative dimers. Our data are compatible with a model where SVP is inactivated as a flowering repressor upon interaction with FUL. This situation would parallel the switch in SVP activity triggered by SVP dimerization with different MADS transcription factors. Thus, it has been proposed that SVP represses flowering during vegetative development, but upon up-regulation of the flowering promoting factor *AGL24* in the SAM, a SVP–AGL24 dimer is formed which is able to activate the expression of *AP1* in early stages of flower development. This model also proposes that once AP1 is present, SVP would be displaced from the interaction with AGL24 to form a complex with AP1 which, in turn, represses the expression of floral organ identity genes, thus ensuring the proper development of floral meristems (Gregis *et al.*, 2006, 2008, 2009).

It is then proposed that SVP would be repressing flowering until other pathways allow the accumulation of SVP interactors such as AGL24 or FUL which, in turn, would form protein complexes with SVP to switch off SVP activity as a flowering repressor.

### The interaction of *FUL* and *FLC* appears to take place at two levels

Our work suggests a major role of *FUL* in promoting flowering on winter ecotypes, as revealed by the enhanced late-flowering phenotype produced by the *ful-2* mutation in the *FRI;FLC* background. Again, this effect is different from that caused by mutations in *SOC1*, since it has been described that *soc1* does not affect the number of rosette leaves of *FRI;FLC* plants or other mutants in the autonomous pathway (Moon *et al.*, 2005). These different effects of *ful* and *soc1* mutations in the *FRI;FLC* background are consistent with the described role of *FLC* in the repression of the photoperiodic stimuli, and the prominent role of *FUL* on flowering promotion under short days. Accordingly, *FUL* loss-of-function delays flowering in the *soc1* and *FRI;FLC* backgrounds. While *FT* and *SOC1* are bona fide targets of FLC negative regulation, no evidence in the literature has been found of FLC regulating *FUL* and, in agreement with that, no binding of FLC on the *FUL* promoter has been detected in ChIP-seq experiments (Deng *et al.*, 2011). Thus, in non-vernalized winter ecotypes, the expression of *FT* and *SOC1* should be repressed by FLC, but *FUL* expression would be regulated independently of FLC, most likely through signals from the age pathway mediated by miR156-targets of the SPL family (Wang *et al.*, 2009; Wu *et al.*, 2009; Yamaguchi *et al.*, 2009).

It has also been observed that *FUL* over-expression was able both to reduce *FLC* expression in the *FRI;FLC* background and to counteract the FLC repressive effect on flowering independently of *FLC* regulation, as revealed by the partial suppression of the 35S::FLC extreme late-flowering phenotype by *FUL* over-expression. These results indicate that FUL could be antagonizing *FLC* at two different levels: by repressing its expression and by competing with FLC activity on its targets. *FLC* repression by FUL might not be direct, as FUL binding on the CArG boxes of the *FLC* promoter could not be detected in ChIP experiments, but it is shown by the observed reduction of FLC::GUS reporter activity in the vegetative tissues of 35S::FUL lines. On the other hand, FUL could also be competing with FLC for SVP dimerization, and thus reduce the repressive effect of FLC–SVP on targets such as *FT* or *SOC1*.

### A model for FUL activity as a modulator of reproductive and meristem identity transitions

With our results on the observed protein–protein interactions as well as the genetic analyses of the *FUL/SVP/SOC1* relationship, we can speculate on a possible mechanism of FUL action to regulate flowering transition in *Arabidopsis* (Fig. 5). During the vegetative phase, both FLC and SVP are able to repress *SOC1* by binding as a heterodimer to the *SOC1* promoter. When FLC and SVP levels are high, as for example in the *FRI;FLC* unvernalized plants, the photoperiodic pathway would be repressed even under long-day conditions. *FUL* expression would increase, gradually responding to signalling from the age pathway. FUL accumulation could then interfere with the FLC–SVP dimer activity, perhaps by displacing SVP from the complex to form an alternative SVP–FUL heterodimer, and thus releasing *SOC1* repression, and/or leading to *SOC1* activation. Upon subsequent SOC1 accumulation, a FUL–SOC1 dimer would form, driving SOC1 protein to the nucleus to maintain its own expression and to activate *LFY* expression and flower initiation, in a possibly redundant manner with AGL24–SOC1 heterodimers.

## Supplementary data

Supplementary data can be found at *JXB* online.

Supplementary Fig. S1. Inflorescence phenotypes of *ful*, *soc1*, and the *ful soc1* double mutant.

Supplementary Fig. S2. Plants used in the ChIP experiments.

Supplementary Fig. S3. Negative controls for BiFC experiments.

Supplementary Table S1. Primers used in this study.

Supplementary Data
