# Supplementary material for: Sequential action of FRUITFULL as a modulator of the activity of the floral regulators SVP and SOC1
Source: J Exp Bot. 2014 Jan 24;65(4):1193–203. doi: 10.1093/jxb/ert482 (PMC3935574; doi:10.1093/jxb/ert482)
Supplement: Supplementary Data [file supp_65_4_1193__index.html]

Sequential action of FRUITFULL as a modulator of the activity of the floral regulators SVP and SOC1 — Supplementary Data 

# Sequential action of *FRUITFULL* as a modulator of the activity of the floral regulators *SVP* and *SOC1*

## Supplementary Data

Data files

**Files in this Data Supplement:**

- Supplementary Data - Supplementary Data
