# Supplementary material for: Sequential action of FRUITFULL as a modulator of the activity of the floral regulators SVP and SOC1
Source: J Exp Bot. 2014 Jan 24;65(4):1193–203. doi: 10.1093/jxb/ert482 (PMC3935574; doi:10.1093/jxb/ert482)
Supplement: Supplementary Data [file supp_ert482_Balanza_SUPPLEMENTARY_MATERIAL_new.pdf]

SUPPLEMENTARY MATERIAL

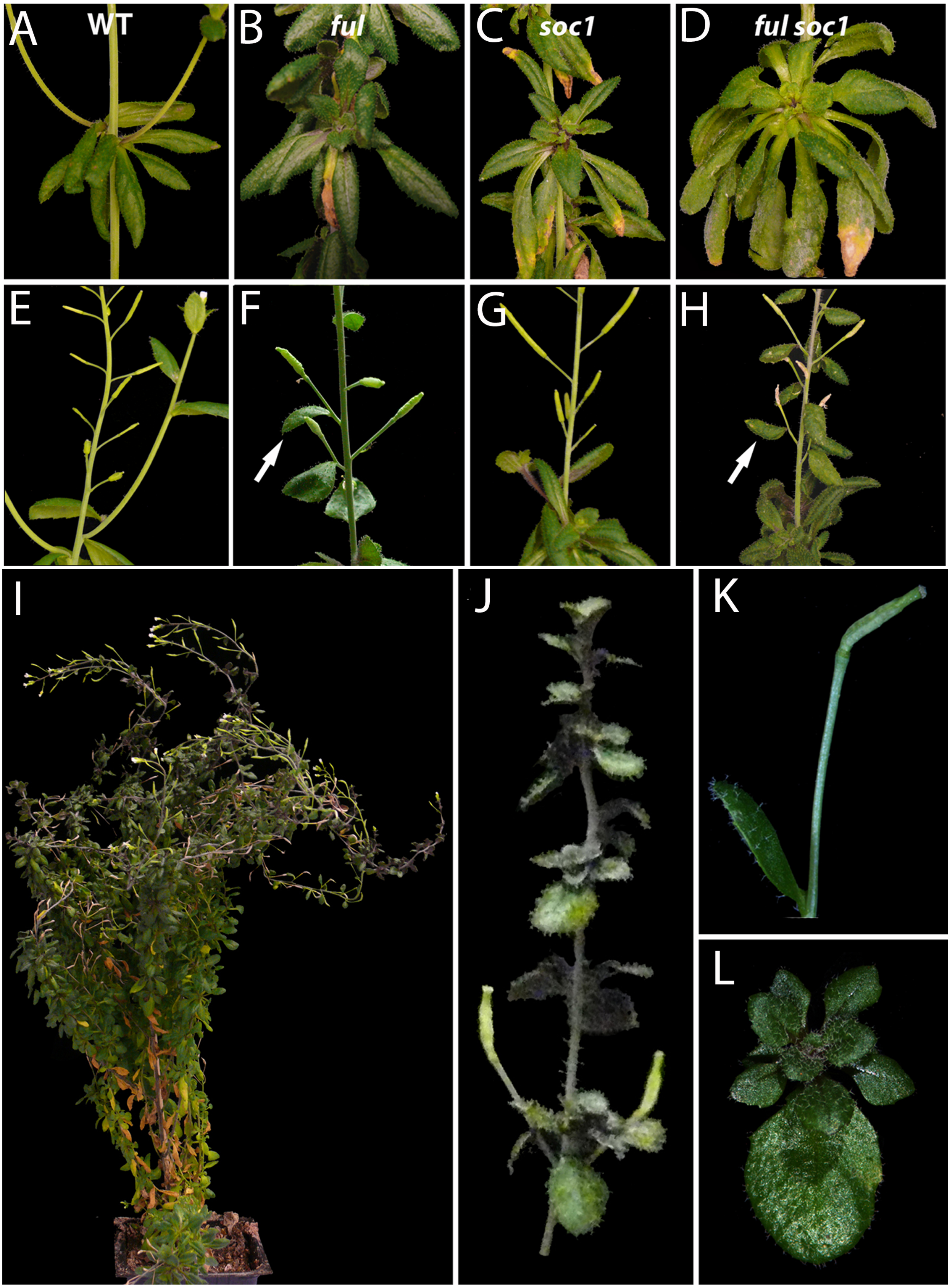

Supplementary figure 1: Inflorescence phenotypes of *ful*, *soc1* and *ful soc1* double mutant.

A-D. Vegetative development of axillary meristems at cauline leaf positions in SD conditions. Axillary meristems in wild type plants produce 2-3 leaves some leaves before the bolting of the secondary inflorescence (A) while *ful*, *soc1* and the double *ful soc1* mutant generate dense aerial rosettes. E-H. Phenotypes of the first flowers produced by the SAM in SD conditions. While wild type (E) and *soc1* (G) mutants develop normal flowers, *ful* (F) and *ful soc1*(H) mutants generate flowers subtended by a leaf-like organ (arrows). I-L. *ful soc1* double mutant grown in LD conditions: Whole plant phenotype (I), reversion of the SAM (J), fruit subtended by a leaf (K) similar to *ful* and *ful soc1* mutants grown in SD conditions (F and H), and aerial rosette developed at the axil of a cauline leaf (L).

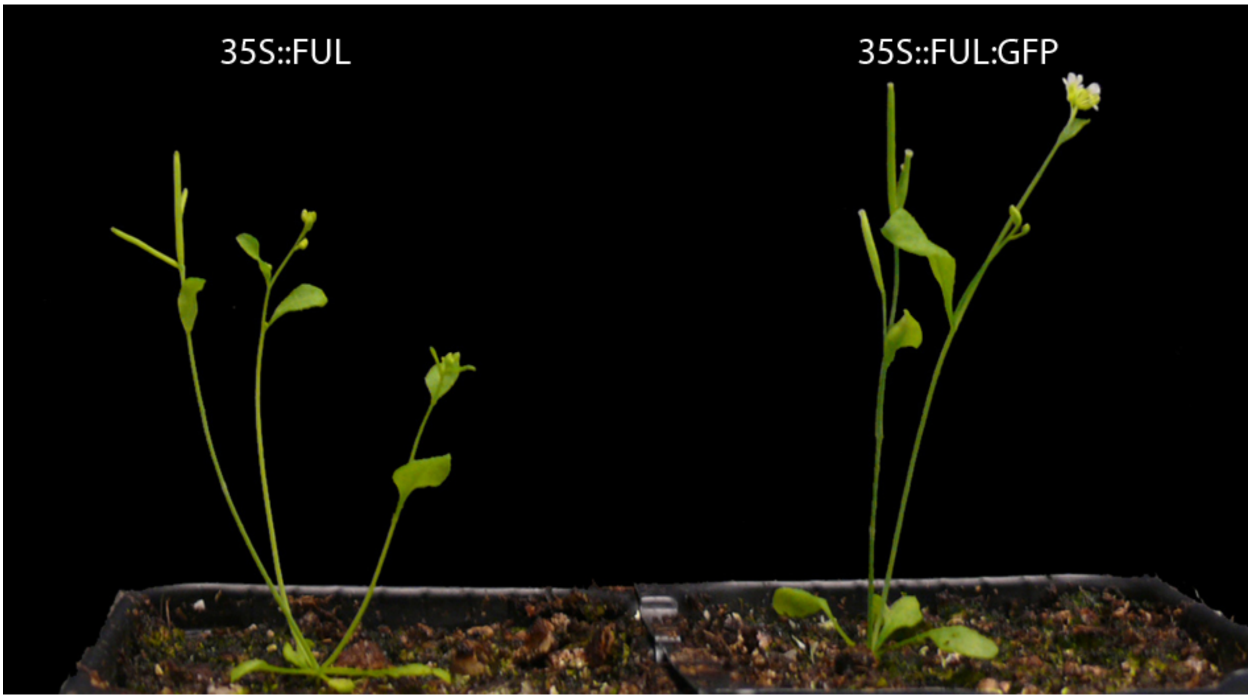

Supplementary figure 2: Plants used in the ChIP experiments.

35S::FUL (right) and 35S::FUL:GFP (left) plants are phenotypically identical, indicating that the GFP fusion does not modify significantly the function of the FUL protein.

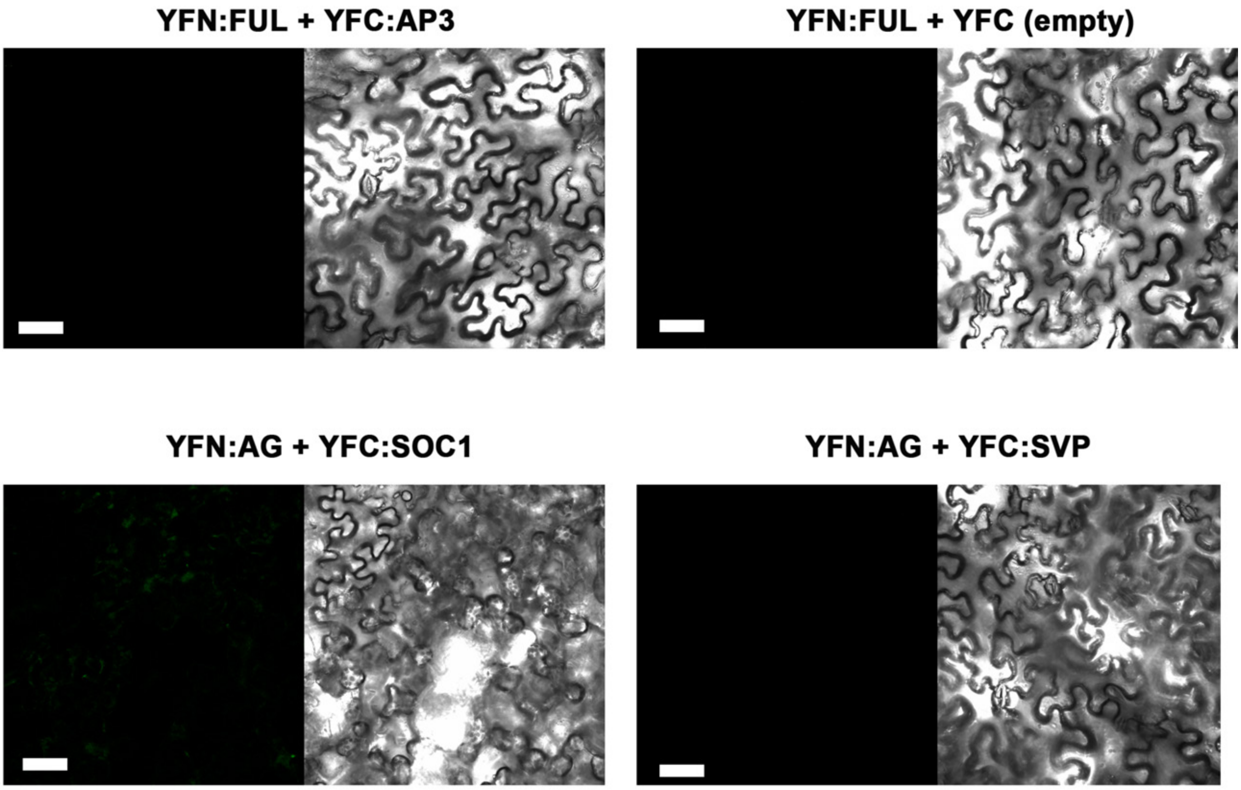

Supplementary figure 3: Negative controls for BiFC experiments.

The YFN:FUL construct was coinfiltrated with a vector with the YFC domain not fused to additional protein domains or with a vector containing a YFC:APETALA3 (YFC:AP3) coding sequence. AP3 is a MADS-box gene and therefore shares structural similarities with SOC1 or SVP. In both cases, no reconstituted fluorescence was observed. Likewise, YFC:SVP and YFC:SOC1 were coinfiltrated with YFN:AGAMOUS (YFN:AG), also a MADS-box gene, and no fluorescence signal was detected.

Supplementary Table 1. Primers used in ChIP experiment

Name	Sequence 5'-3'		Position from ATG	
LFY-1f	CTATACGACGTCGTTTGAAAGGGATCC	forward	-2311 a -2285	LEAFY
LFY-1r	GCGTTTATATCTTCTCGGTCAGCCCA	reverse	-2205 a -2180	LEAFY
LFY-2f	TATCTTCCCCTAACAATACTTCCAAAGC	forward	-1045 a -1018	LEAFY
LFY-2r	TCTTTGCAGAAGCCCGATAAGTTACT	reverse	-940 a -915	LEAFY
SOC1-1f	TATATCGGGAGGAGGACCACAC	forward	-1805 a -1784	SOC1
SOC1-1r	ATCCATACAGATTTTCGGACCT	reverse	-1703 a -1682	SOC1
SOC1-2f	TGGACGCTTGAAACCTCATCCT	forward	-1028 a -1007	SOC1
SOC1-2r	GGGAGGGAAAAAGATGTGTATG	reverse	-936 a -915	SOC1
SOC1-3f	GCAAAAGAAGTAGCTTTCCTCG	forward	-752 a -730	SOC1
SOC1-3r	AGCAGAGAGAGAAGAGACGAGTG	reverse	-678 a -657	SOC1
SOC1-4f	GGATGCAACCTCCTTTCATGAG	forward	-396 a -375	SOC1
SOC1-4r	ATATGGGTTTGGTTTCATTTGG	reverse	-339 a -318	SOC1
SOC1-5f	ATCACATCTCTTTGACGTTTGCTT	forward	310 a 333	SOC1
SOC1-5r	GCCCTAATTTTGCAGAAACCAA	reverse	359 a 380	SOC1
SOC1-6f	TGTTTCAGACATTTGGTCCATTTG	forward	940 a 963	SOC1
SOC1-6r	AGTCTTGTACTTTTTCCCCCTATTTTAG	reverse	1029 a 1056	SOC1
ACT f	CGTTTCGCTTTCCTTAGTGTTAGCT	forward	-657 a -633	ACTIN7
ACT r	AGCGAACGGATCTAGAGACTCACCTTG	reverse	-550 a -524	ACTIN7
UBQ10f	GGCCTTGTATAATCCCTGATGAATAAG	forward	1446 a 1472	UBIQUITIN10
UBQ10r	AAAGAGATAACAGGAACGGAAACATAGT	reverse	1479 a 1506	UBIQUITIN10

Primers used for qRT-PCR experiments

TIP41-like	GTGAAAACTGTTGGAGAGAAGCAA	forward		TIP41-like
TIP41-like	TCAACTGGATACCCTTTCGCA	reverse		TIP41-like
LFYf	TCCACTGCCTAGACGAAGAAGC	forward		LFY
LFYr	TCCCAGCCATGACGACAAGC	reverse		LFY
FLCf	CGTCGCTCTTCTCGTCGTCTC	forward		FLC
FLCr	TTCGGTCTTCTTGGCTCTAGTCAC	reverse		FLC
